# Effects of different lower limb movement velocities on trunk muscle activity and dynamic stability in the Sahrmann core stability test

**DOI:** 10.1371/journal.pone.0333004

**Published:** 2025-09-24

**Authors:** Kohei Yoshikawa, Matsuri Hashimoto, Rina Murata, Yuta Murata, Takumi Jiroumaru, Noriyuki Kida

**Affiliations:** 1 Department of Rehabilitation, Kanazawa Orthopaedic and Sports Medicine Clinic, Ritto, Shiga, Japan; 2 Graduate School of Science and Technology, Kyoto Institute of Technology, Kyoto, Kyoto, Japan; 3 Department of Rehabilitation, Furu Clinic, Koka, Shiga, Japan; 4 Department of Physical Therapy, School of Health Science, Bukkyo University, Kyoto, Kyoto, Japan; 5 Faculty of Arts and Sciences, Kyoto Institute of Technology, Kyoto, Kyoto, Japan; Iran University of Medical Sciences, IRAN, ISLAMIC REPUBLIC OF

## Abstract

Core stability is essential for both performance and rehabilitation, yet standardized movement velocity criteria for the Sahrmann Core Stability Test (SCST) remain undefined. This study aimed to analyze how lower limb movement velocity affects trunk muscle activation patterns across movement phases and sides during SCST Level 3 in a supine position. SCST Level 3 was selected because it challenges both deep and superficial muscles without being too difficult for healthy participants. Sixteen healthy adult males, recreationally active but not involved in a training program at the time of recruitment, performed leg-lowering movements at three velocities: slow (5 s), medium (3 s), and fast (1 s). Surface electromyography recorded activation in the internal oblique/transversus abdominis (IO/TrA), external oblique (EO), and rectus abdominis (RA). Dynamic stability was simultaneously monitored using an inflatable pressure-biofeedback unit positioned at L4–L5 and inflated to 40 mmHg; participants maintained the pressure within ±10 mmHg throughout each trial. Muscle activity was analyzed across five phases: 500 ms and 250 ms before movement (pre-500 ms, pre-250 ms), and three post-movement phases (post 1, post 2, post 3). A three-way repeated-measures ANOVA (factors: velocity, movement phase, and laterality) revealed significant velocity-phase interactions for all muscles (*p* < 0.05; IO/TrA: *η*^*2 *^= 0.170; EO: *η*^*2*^ = 0.198; RA: *η*^*2*^ = 0.153), indicating that velocity affected the temporal activation pattern, particularly during the middle phase of the movement. Faster movements led to a rapid increase in IO/TrA, EO, and RA activity at post 2, whereas slower movements showed a gradual increase. Activation levels converged across conditions by the final phase, with no significant differences between deep and superficial muscles or between sides. These findings suggest SCST Level 3 consistently elicits high trunk muscle activation regardless of velocity. However, faster movements may provide valuable insights into trunk stabilization during mid-movement for clinical evaluations.

## Introduction

Assessing and improving core stability is essential for enhancing sports performance and preventing musculoskeletal disorders. Core stability involves maintaining spinal and pelvic alignment through coordinated activation of deep and superficial muscles, ensuring efficient limb movements and minimizing injury risk [1–4]. Reduced core stability has been reported to increase the risk of musculoskeletal disorders, including lower back pain and decrease athletic performance [5,6]. While generally categorized by muscular function (stabilization through deep and superficial muscle activity), structural stability (maintenance of proper spinal and pelvic alignment), and dynamic stability (postural control in response to external loads and limb movements) [1,4,7–9], core stability lacks a single, universally accepted definition [10].

Assessment methods are broadly classified as static or dynamic. Static tests, such as the plank and side bridge test, evaluate fundamental endurance-based support capacity in postural control [11]. However, real-life and sports activities involve dynamic challenges, requiring the core to withstand significant instantaneous loads, such as multidirectional upper and lower limb movements or landing impacts. Therefore, assessment of core stabilization under dynamic conditions has been increasingly emphasized [12]. Dynamic assessments better capture the real-world demands for core muscles to contract quickly and in a coordinated motion to stabilize the spine and pelvis in response to limb movements or external loads [2]. Studies show that the transversus abdominis (TrA) contracts in a feedforward manner before limb movement initiation, stabilizing the spine [13–17]. This mechanism is thought to help absorb inertial forces generated by limb movements, thereby minimizing spinal displacement and maintaining core stability.

The Sahrmann Core Stability Test (SCST) is a widely used dynamic core stability assessment that measures a subject’s ability to maintain spinal and pelvic alignment while lowering the lower limbs in a supine position. Pressure sensors monitor lumbar and pelvic stability, providing quantitative insight into core muscle activation, which is difficult to capture through static assessments [12,18]. In the SCST, movement velocity critically alters inertial and gravitational forces, potentially affecting muscle activation patterns. Despite its widespread use, a significant clinical challenge in the SCST is the complete absence of standardized movement velocity guidelines (“slow,” “medium,” or “fast”), leading to considerable variability in test execution across instructors or subjects. Indeed, a recent EMG investigation confirmed that no standardized velocity guideline currently exists for the test, allowing participants to perform it at a self-selected velocity [12]. This critical lack of standardization underscores the need to thoroughly investigate the effects of movement velocity on core muscle activation. Given prior findings on velocity-dependent TrA activation [14,17,19,20], movement velocity may significantly influence core muscle activation patterns.

The influence of movement velocity on core muscle activation has been demonstrated in several studies. It is known that faster limb movements elicit earlier activation of core muscles, such as TrA, to provide anticipatory postural adjustments [19]. This principle extends to various contexts; for instance, TrA has been reported to activate first in response to hip flexion and extension during lower limb movements, with findings varying across postures such as supine [17] and walking [20], suggesting that velocity influences core muscle control across different contexts [14]. In particular, anticipatory TrA contraction is considered essential for maintaining core stability during rapid limb movements [13–17]. Crucially, faster limb movements impose greater inertial (and gravitational) loads on the lumbopelvic region, thereby requiring a more immediate and robust anticipatory stabilizing response from trunk muscles, particularly the internal oblique/transversus abdominis (IO/TrA) complex, to limit spinal displacement and maintain core stability [13–17]. Supine active straight-leg-raise paradigms further show bilateral abdominal engagement that is sensitive to external stabilization (pelvic belt), task loading, and movement phase [21–23]. Importantly, recent work indicates that altering lower limb raising velocity can modulate abdominal activation magnitude, suggesting that velocity is a clinically meaningful parameter in supine lumbopelvic control tests [24]. More pertinently for supine tasks, studies have investigated trunk muscle thickness changes during hip extension exercises in supine positions [25], and analyzed kinematics and electromyography activity during lower limb raising from the supine position, observing varied muscle activation patterns [26]. These findings suggest that differences in velocity and posture may alter core muscle activation patterns. However, its effects on muscle activation timing, intensity, and asymmetry during supine dynamic stability tests remain unclear. Therefore, elucidating the effects of movement velocity is essential for standardizing the SCST and optimizing exercise guidance. Understanding these velocity-dependent patterns is crucial for clinicians to accurately assess functional deficits, identify specific needs for rehabilitation, and tailor exercise prescriptions to meet diverse athletic or daily living demands.

This study focused on SCST Level 3 to investigate how movement velocity affects core muscle activation timing, intensity, and asymmetry. Level 3 can be performed without difficulty by healthy participants and has been shown to elicit relatively high activation of both deep and superficial trunk muscles [12]. For these reasons, we selected Level 3 for the present study. We formulated specific a priori hypotheses: faster leg-lowering velocity would augment deep stabilizer activity such that IO/TrA %MVC would be higher in the anticipatory pre-250 ms window (the pre-500 ms window serving as a low, velocity-invariant baseline); after movement onset, IO/TrA, EO, and RA amplitudes would scale with velocity and diverge most during the middle third of the descent (post 2), when inertial demand peaks, and that this divergence would persist into the final third (post 3), with the fast condition maintaining the highest level of trunk muscle activation at task completion; and because only the right leg is lowered, IO/TrA and EO would exhibit greater activation on the ipsilateral side during early-to-mid movement (post 1–post 2), whereas side differences in RA would be minimal. Findings from this study are intended to refine SCST application for core-stability assessment and exercise prescription.

## Materials and methods

### Participants

A total of 16 healthy adult males (24.9 ± 5.0 years, 171.8 ± 7.2 cm, 64.6 ± 6.2 kg), all of whom were right-leg dominant as determined by their preferred kicking leg, participated in this study. All participants were recreationally active but not involved in a specific training program at the time of recruitment. We restricted enrollment to healthy young males to reduce between‑participant variability related to sex, age, and musculoskeletal status and to isolate the effects of leg‑lowering velocity on trunk muscle activation; this homogeneous sampling improves internal validity but limits generalizability. Exclusion criteria included a history of pain, significant postural abnormalities, or neurological or respiratory disorders. Written informed consent was obtained from every participant. The study was approved by the Ethics Committee of Kanazawa Orthopedic Sports Medicine Clinic (Kanazawa-OSMC-2024–010) and conducted in accordance with the Declaration of Helsinki. The recruitment period for this study was from 5 October 2024–20 December 2024.

A power analysis using G*Power software (version 3.1.9.4, Germany) determined the required sample size. We planned a repeated-measures ANOVA with three within-subject velocity levels and adopted a moderate-to-large planning effect (Cohen f = 0.35). This value was selected between Cohen’s conventional medium (0.25) and large (0.40) benchmarks and judged physiologically meaningful because comparable core-stability and supine lower-limb electromyography studies report clear condition differences in trunk-muscle activation but do not provide standardized effect sizes. With α = 0.05, power (1–β) = 0.80, correlation among repeated measures = 0.50, and non-sphericity correction ε = 1.0, the required sample size was 15. To account for potential dropouts and data variability, 16 participants were recruited.

### Kinematic data acquisition

Kinematic data were collected using a wireless electromyography (EMG) and accelerometer system (LP-WS1222, LOGICAL PRODUCT Co., Ltd., Fukuoka, Japan). The accelerometer, placed on the anterior mid-thigh of the right femur, recorded movement when the Z-axis signal exceeded ±3 standard deviations (SD) from the mean baseline activity recorded in the initial posture. Movement termination was defined as the point when the predefined movement for each condition was completed.

### Electromyography (EMG) recording

**Fig 1:**
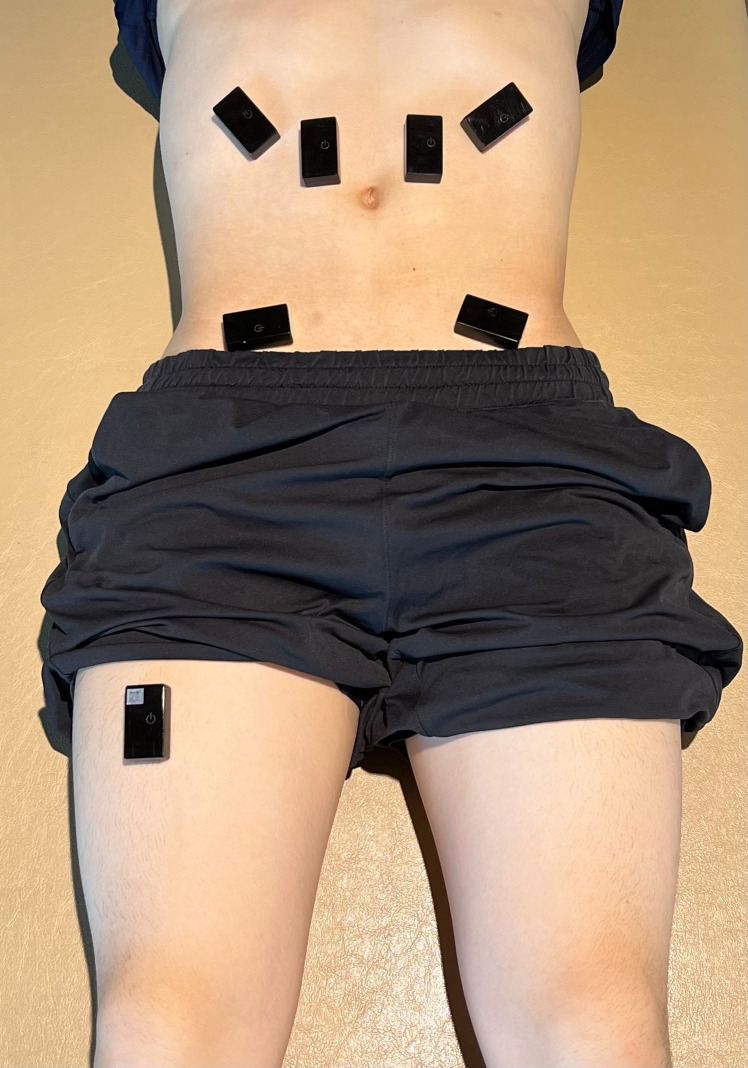
Placement of surface electromyography electrodes on abdominal muscles and an accelerometer on the thigh. This figure presents the placement of sEMG electrodes and the accelerometer used in the study. Electrodes were positioned as follows: rectus abdominis (RA), 1 cm superior and 2 cm lateral to the umbilicus; external oblique (EO), 1 cm superior to the horizontal umbilical line and 1 cm lateral to the RA border; and internal oblique/ transversus abdominis (IO/TrA), 1 cm medial to the anterior superior iliac spine (ASIS) and 0.5 cm inferior to the ASIS line. Electrodes were placed on both the right and left sides, giving a total of six sEMG channels. The accelerometer was positioned on the anterior mid-thigh of the right femur.

Skin preparation for EMG recording involved shaving and reducing skin impedance to <5 kΩ and drying the skin before placing wireless EMG sensors (LP-WS1221, LOGICAL PRODUCT Co., Ltd., Fukuoka, Japan) with a 20-mm interelectrode distance. Electrode placements for the rectus abdominis (RA) were positioned 1 cm above and 2 cm lateral to the umbilicus [27], 1 cm above the horizontal umbilical line and 1 cm lateral to the RA border for the external oblique (EO) [28], 1 cm medial to the anterior superior iliac spine (ASIS) and 0.5 cm below the ASIS line for the internal oblique and transversus abdominis (IO/TrA) [29]. All electrodes were applied bilaterally, resulting in six recording sites (right and left RA, EO, and IO/TrA) (Fig 1). These placements were based on previous studies to accurately capture combined IO/TrA muscle activity, and real-time ultrasound verification confirmed that the electrode sites corresponded to the validated positions reported earlier [29–32]. Ultrasound confirmed IO/TrA muscle fiber orientation to minimize overlap with the EO. To minimize crosstalk, special attention was given to electrode placement, interelectrode distance, and alignment with muscle fibers [33]. EMG signals were sampled at 1,000 Hz and stored in the internal memory of the wireless sensor system.

### Experimental task

Dynamic core stability was assessed using the SCST Level 3, which has high reliability (intraclass correlation coefficient = 0.95, measurement error = 7.7%) [34]. The test comprises five levels, with Level 3 selected for this study as it engages all major core muscles, including the TrA, RA, IO, and EO. This level is considered particularly valuable for assessing dynamic core stability [12].

An inflatable pressure biofeedback unit (PBU; Stabilizer Pressure Biofeedback Unit, REHABMEDIC, Barcelona, Spain) was positioned around the L4–L5 region of the lumbar spine. Use of a PBU to cue and monitor lumbopelvic alignment during core stability and motor control assessments is well established in clinical and research settings, with studies reporting acceptable intra-/interexaminer and inter-rater reliability across lower limb control tests and abdominal drawing-in protocols in populations with and without low back pain [35–37]. Participants lay supine with knees flexed and lifted to 90° hip and knee flexion, followed by the contralateral leg, maintaining both legs in position. The PBU was inflated to 40 mmHg, and participants were instructed to maintain lumbar alignment by keeping the pressure within 40 ± 10 mmHg; this pressure maintenance served as an indicator of dynamic lumbar stability throughout the task. Throughout each trial, an experienced physical therapist visually monitored the PBU gauge in real time in accordance with the clinical SCST procedure. If pressure drifted outside the ± 10 mmHg tolerance (<30 or >50 mmHg), the trial was stopped, the participant was re-cued, and the trial was repeated; only trials that remained within tolerance for the full movement (visual confirmation) were retained for analysis. Because the PBU signal was not digitally recorded, we could not compute pressure-time indices, error counts, or across-trial pressure variability (e.g., standard deviation).

**Fig 2:**
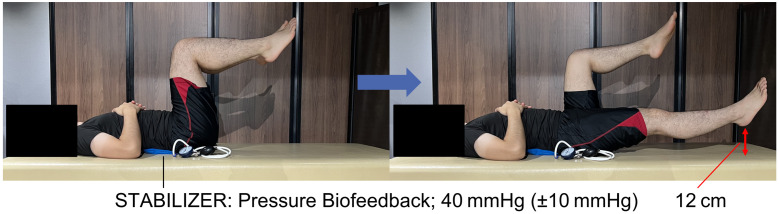
Experimental setup and lower limb lowering task performed under three velocity conditions. This figure presents the testing posture and movement sequence used in the study. Participants lay supine with both hips and knees flexed to 90°, with an inflatable pressure-biofeedback unit (PBU) secured around the L4–L5 region and inflated to 40 mmHg (starting position). While keeping the PBU reading within 40 ± 10 mmHg to maintain lumbar alignment, each participant lowered the right leg until the heel hovered 12 cm above the floor. The descent was executed at three preset velocities—slow (5 s), medium (3 s), and fast (1 s)—administered in randomized order.

From this position, all participants gradually lowered the right leg until the heel reached 12 cm above the ground while maintaining the preset pressure (Fig 2). The test was performed under three velocity conditions: slow (5 s for descent), medium (3 s for descent), and fast (1 s for descent). To minimize fatigue and learning effects, the order of conditions was randomized. A three-minute rest period was provided between trials. Each condition was performed twice, and the mean of the two trials was used for analysis.

Maximum voluntary isometric contraction (MVIC) tests were performed for normalization. Participants performed two five seconds MVICs against manual resistance in a supine position with adequate rest between sets, with the higher of the two trials used for normalization. RA MVC was measured during a maximal curl-up against manual resistance; EO MVC during trunk rotation to the contralateral side against manual resistance; and IO/TrA MVC during trunk rotation to the ipsilateral side against manual resistance. Each task was performed in both sitting and crook-lying positions, and the posture that produced the greater EMG activity for each participant was retained for analysis. Participants were instructed to contract the target muscles as forcefully as possible in every trial [38,39].

### Data analysis

The definitions of movement onset and termination were based on the Z-axis component of the accelerometer. Movement onset was identified as the point at which the signal exceeded ±3 SD from the baseline mean in the starting position. Movement termination was defined as the point at which the predefined movement duration (5 s, 3 s, or 1 s) had elapsed.

EMG data were analyzed from 500 ms before movement onset until termination. Raw signals were band-pass filtered (20–500 Hz), full-wave rectified, and processed using a 50-ms root mean square (RMS) moving window. Coefficients of variation (CV = SD/ mean × 100%) were computed for each muscle-velocity-phase-side cell to quantify inter-individual variability. Log-transformed %MVC values were also screened for outliers using a robust z-score criterion (|z| > 3.0); none were detected. The movement phase was divided into five intervals: pre-500 ms (500 ms before movement onset), pre-250 ms (250 ms before movement onset), post 1 (first third of the movement phase), post 2 (middle third of the movement phase), and post 3 (final third of the movement phase) (Table 1). Selection of the two pre-movement intervals was guided by previous reports of feed-forward trunk muscle activity. Anticipatory postural adjustments (APAs) emerge 50–200 ms before limb motion, whereas anticipatory synergy adjustments (ASAs) can begin as early as 250–300 ms when the timing or direction of the forthcoming action is predictable [14,29,40]. We therefore set the pre-250 ms window to encompass this entire anticipatory cascade. The longer pre-500 ms window was treated as the resting baseline, because trunk muscle EMG recorded ≥ 400 ms before movement remains at or very near resting levels [2]; visual inspection of our traces confirmed that muscle activity throughout the −500 to −250 ms window stayed within this noise band. Muscle activation was expressed as a percentage of MVIC (%MVC). Data processing was conducted using MATLAB R2024a (The MathWorks, Inc., Natick, MA, USA).

**Table 1:** Phase segmentation for each movement velocity condition (slow, medium, fast).

	pre 500ms (ms)	pre 250ms (ms)	post 1 (ms)	post 2 (ms)	post 3 (ms)
slow (5 s)	pre 500 ~ 251	pre 250 ~ 1	post 0 ~ 1666	post 1667 ~ 3333	post 3334 ~ 5000
medium (3 s)	pre 500 ~ 251	pre 250 ~ 1	post 0 ~ 1000	post 1001 ~ 2000	post 2001 ~ 3000
fast (1 s)	pre 500 ~ 251	pre 250 ~ 1	post 0 ~ 333	post 334 ~ 666	post 667 ~ 1000

The pre-500 ms and pre-250 ms phases represent time intervals before movement onset, while Post 1, Post 2, and Post 3 correspond to the first, middle, and final thirds of the movement phase, respectively. The time ranges for each phase vary based on the movement velocity condition.

### Statistical analysis

%MVC data were log-transformed (log10) for normality and confirmed to follow a normal distribution using the Shapiro–Wilk test. A three-way repeated-measures ANOVA was conducted to examine the effects of movement velocity (slow, medium, fast), movement phase (pre-500 ms, pre-250 ms, post 1, post 2, post 3), and side (right, left). If sphericity was violated, the Greenhouse–Geisser correction was applied. Significant effects or interactions were analyzed using Bonferroni-corrected multiple comparisons. Statistical analyses were performed using SPSS ver. 29.0.2 (IBM Corp., Armonk, NY, USA), with p < 0.05 considered statistically significant.

## Results

**Fig 3:**
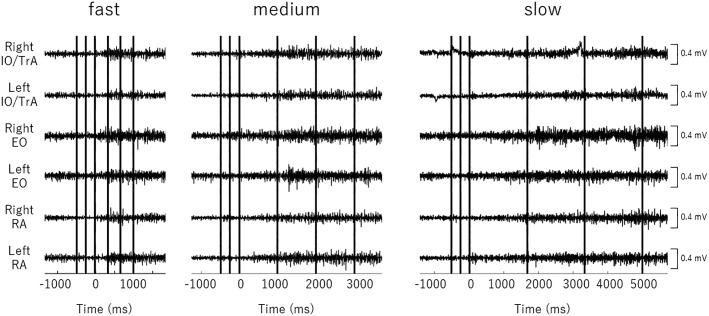
Representative raw surface electromyography (sEMG) waveforms This figure presents representative raw surface electromyography (sEMG) waveforms from a single participant during the Sahrmann Core Stability Test (SCST) Level 3, performed at three different movement velocities (slow, medium, and fast). The vertical axis represents muscle activity amplitude (mV), while the horizontal axis denotes time (ms), with 0 ms defined as the onset of limb movement. Waveforms are divided into five phases: pre-500 ms (−500 to −251 ms), pre-250 ms (−250 to −1 ms), and three post-movement phases corresponding to the first third (post 1), middle third (post 2), and final third (post 3) of the movement. Each phase is marked by solid lines. Muscle abbreviations: RA (rectus abdominis), EO (external oblique), IO/TrA (internal oblique/transversus abdominis). These waveforms illustrate the performance of a single participant and do not represent the group average.

Fig 3 and Table 2 present the results for each muscle. A three-way repeated-measures ANOVA (factors: velocity, phase, and side) revealed a significant interaction between velocity and phase for all three muscles (IO/TrA: **p* *= 0.004, *η*^*2*^* *= 0.170; EO: **p* *= 0.016*η*^*2*^* *= 0.198; RA: *p* = 0.031, *η*^*2*^* *= 0.153) (Table 3). A significant main effect of phase was also observed (Table 4), while velocity, side, and other interactions showed no significant effects.

**Table 2:** Surface electromyography amplitudes expressed as a percentage of maximal voluntary contraction (%MVC) for trunk muscles across velocities and time windows.

Muscles	Velocity	Side	pre 500ms (%)	pre 250ms (%)	post 1 (%)	post 2 (%)	post 3 (%)
IO/TrA	slow (5 s)	Right	9.06 ± 6.17	9.80 ± 8.27	12.48 ± 6.62	16.41 ± 6.32	18.32 ± 9.19
		Left	6.93 ± 1.54	7.17 ± 1.99	9.71 ± 2.53	12.90 ± 4.05	15.14 ± 4.72
	medium (3 s)	Right	8.60 ± 4.39	7.99 ± 3.41	11.72 ± 3.77	16.04 ± 5.43	18.49 ± 7.17
		Left	7.29 ± 2.22	7.32 ± 2.65	10.30 ± 3.54	13.36 ± 3.58	15.30 ± 4.38
	fast (1 s)	Right	8.59 ± 5.32	8.06 ± 4.07	12.12 ± 7.26	20.11 ± 7.28	19.99 ± 7.23
		Left	6.85 ± 2.08	7.22 ± 2.25	9.35 ± 3.87	15.43 ± 4.83	15.57 ± 5.02
EO	slow (5 s)	Right	16.80 ± 6.09	16.08 ± 5.39	22.44 ± 8.35	30.69 ± 11.96	34.05 ± 12.02
		Left	16.13 ± 8.35	16.99 ± 9.64	21.48 ± 11.76	26.95 ± 13.00	31.30 ± 12.97
	medium (3 s)	Right	16.70 ± 4.60	17.98 ± 6.65	23.58 ± 9.75	30.46 ± 10.97	34.37 ± 12.22
		Left	16.27 ± 7.94	16.27 ± 7.82	22.17 ± 10.56	29.21 ± 15.59	32.31 ± 15.70
	fast (1 s)	Right	16.16 ± 5.42	15.95 ± 6.41	21.58 ± 6.72	34.53 ± 12.12	37.32 ± 11.65
		Left	15.60 ± 8.73	15.60 ± 10.80	21.24 ± 11.68	32.32 ± 20.21	33.30 ± 16.77
RA	slow (5 s)	Right	9.78 ± 5.36	9.11 ± 5.05	13.28 ± 6.75	18.11 ± 8.65	21.31 ± 9.71
		Left	9.00 ± 4.87	8.71 ± 4.62	13.27 ± 6.68	18.53 ± 9.80	21.10 ± 10.73
	medium (3 s)	Right	9.41 ± 4.96	9.11 ± 4.97	12.98 ± 5.63	17.63 ± 7.26	20.60 ± 8.52
		Left	9.85 ± 5.20	9.01 ± 4.81	13.09 ± 5.43	17.95 ± 8.10	21.14 ± 9.38
	fast (1 s)	Right	9.77 ± 6.17	9.06 ± 6.26	13.18 ± 8.11	21.52 ± 12.80	22.02 ± 10.98
		Left	9.01 ± 4.86	8.32 ± 5.57	11.13 ± 6.56	20.55 ± 10.32	21.74 ± 10.40

Values are presented as mean ± SD for the internal oblique/ transversus abdominis (IO/TrA), external oblique (EO), and rectus abdominis (RA). Data are shown for three lowering velocities—slow (5 s), medium (3 s), and fast (1 s)—with right- and left-side values reported separately across five times windows (pre-500 ms, pre-250 ms, post 1, post 2, post 3). CVs (coefficients of variation) ranged from 22% to 84%; values > 70% occurred only in pre-movement windows (mean %MVC < 10%), while post-movement phases showed CVs of 26–60%.

**Table 3:** Three-way repeated-measures ANOVA: Interaction effects. Factors: Velocity, Phase, and Side on log10-transformed %MVC data for each muscle (IO/TrA, EO, RA).

	Interaction											
	Velocity × Phase			Velocity × Side			Phase × Side			Velocity × Phase × Side		
	*F*	*p*	*η* ^*2*^	*F*	*p*	*η* ^*2*^	*F*	*p*	*η* ^*2*^	*F*	*p*	*η* ^*2*^
IO/TrA	3.068	0.004*	0.170	0.525	0.597	0.034	1.524	0.236	0.092	0.764	0.635	0.048
EO	3.704	0.016*	0.198	1.665	0.206	0.100	0.483	0.642	0.031	1.783	0.136	0.106
RA	2.718	0.031*	0.153	0.970	0.391	0.061	0.970	0.391	0.061	1.156	0.335	0.072

F = F-value; p = p-value; η² = partial eta-squared. Significance was set at p < 0.05.

**Table 4:** Three-way repeated-measures ANOVA: Main effects. Main effects from the same three-way repeated-measures ANOVA (Velocity, Phase, Side) presented in Table 3.

	Main effect								
	Velocity			Phase			Side		
	*F*	*p*	*η* ^*2*^	*F*	*p*	*η* ^*2*^	*F*	*p*	*η* ^*2*^
IO/TrA	0.160	0.853	0.011	82.917	<.001*	0.847	2.656	0.124	0.150
EO	0.548	0.584	0.035	96.443	<.001*	0.865	0.582	0.457	0.037
RA	0.211	0.737	0.014	127.107	<.001*	0.894	0.014	0.907	0.001

F = F-value; p = p-value; η² = partial eta-squared. Significance was set at p < 0.05.

Post hoc Bonferroni analysis showed no significant changes in muscle activity between pre-500 ms and pre-250 ms across all three muscles (IO/TrA, EO, RA) under any movement velocity conditions (slow, medium, fast). However, from pre-250 ms to post 1, EO and RA activity significantly increased under all three velocity conditions (p < 0.05), while IO/TrA activity increased only under slow and medium velocity conditions (p < 0.05) with no change under the fast condition.

From post 1 to post 2, all three muscles exhibited significant increases across all velocity conditions (p < 0.05). Under the fast condition, activity increased substantially, leading to significantly higher IO/TrA and EO activation at post 2 compared to the slow condition (p < 0.05). Between post 2 and post 3, significant increases continued for all three muscles under slow and medium conditions (p < 0.05), but not under the fast condition, where activation had already markedly increased by post 2 and plateaued subsequently.

When benchmarked against the clinically meaningful threshold of 10% MVC [41], EO and RA exceeded this level under all velocity conditions from Post 1 onward. For IO/TrA, the left side failed to reach 10% MVC at Post 1 under the slow and fast velocities, but all other conditions surpassed the threshold; from Post 2 onward, IO/TrA exceeded 10% MVC across all velocities.

By post 3 (final phase of movement), differences in all three muscle activities across movement velocities had disappeared, indicating convergence of activation levels regardless of movement velocity.

All individual-level values underlying the analyses are provided in S1 Table (CSV).

## Discussion

This study examined how lower-limb lowering velocity modulates trunk muscle activity during SCST Level 3. A three-way repeated-measures ANOVA confirmed a significant velocity × phase interaction for IO/TrA, EO, and RA (p < 0.05), whereas side showed no main or interaction effects, indicating that velocity chiefly alters temporal recruitment rather than left–right symmetry.

We did not observe anticipatory increases in any muscle between −500 and −250 ms before movement onset; therefore, we refrain from claiming anticipatory activation in this paradigm. Importantly, lack of observation does not imply absence: anticipatory activity may have been potentially present but undetected due to methodological limitations. First, the SCST Level 3 start posture (90° hip and knee flexion) already imposes substantial baseline co-contraction (6–10% MVC for deep muscles; 8–16% MVC for superficial muscles), a level sufficient to stabilize the lumbar spine and known to blur individual anticipatory patterns [12,41–43]. Second, our analysis using relatively wide 250 ms windows may have lacked sensitivity to detect very brief (<100 ms), low-amplitude anticipatory bursts previously identified [13–15,17].

Consequently, velocity-related changes in muscle activation were more evident during the post-initiation phase than in the pre-movement phase, suggesting that under our protocol adjustments made during movement execution were more prominent than anticipatory control before movement onset.

Rapid lowering elicited an early-phase activation surge, whereas slower velocities produced a more progressive late-phase rise; despite these temporal differences, all three velocities converged on a comparable level of co-contraction by the task’s end, indicating that terminal lumbopelvic stability is essentially velocity-independent. This finding partially contradicted our hypothesis that the fast condition would sustain the highest terminal muscle activation due to greater inertial demand. One plausible explanation is that once co-contraction exceeds a certain threshold (approximately 10% MVC), further increases offer diminishing returns in terms of spinal stability, resulting in a ceiling effect that masks between-velocity differences at the task’s end.

Although side did not reach statistical significance, descriptive analysis indicated that IO/TrA and EO activity tended to be greater on the ipsilateral (right) side during early to mid-phases (post 1 to post 2), consistent with the unilateral nature of the leg-lowering task. In contrast, RA showed minimal side-related differences, supporting our hypothesis of muscle-specific asymmetry.

The earlier mid-phase surge in activation, particularly under the fast condition, suggests transient stability demands that might prove functionally relevant during rapid deceleration or directional-change tasks. Recent motor-control models emphasize that trunk stability emerges from low-dimensional muscle synergies. Experimental synergy analyses using non-negative matrix factorization have repeatedly identified two dominant patterns during unilateral leg tasks: an abdominal bracing pattern, characterized by near-synchronous co-activation of IO/TrA, EO, and RA that stiffens the abdominal wall, and a posterior-chain pattern in which erector spinae and multifidus carry the largest weights [44,45]. The simultaneous rise in IO/TrA and EO activity observed in the mid-to-late phases of our protocol is compatible with recruitment of the abdominal bracing pattern. This interpretation is further supported by findings in walking: deep muscles such as the TrA maintain sustained activity during slow gait and synchronize more closely with superficial muscles as velocity increases [20], a pattern that mirrors our velocity-dependent results.

From a functional perspective, deep muscles such as IO/TrA stabilize individual spinal segments, while superficial muscles such as EO and RA contribute to torque generation and gross trunk movements like rotation and flexion [46–48]. We hypothesized that fast lowering would produce earlier and greater activation of deep stabilizers (IO/TrA) than superficial muscles (EO, RA), based on their roles in segmental versus gross trunk control. However, activity in IO/TrA, EO and RA all rose in parallel from Post 1 to Post 2 across all velocities, with IO/TrA and EO showing the largest increase under the fast condition, and only the slow and medium conditions continuing to increase into Post 3.

These results indicate that, in the supine Level 3 task, deep and superficial muscles engage simultaneously in a cooperative bracing strategy rather than exhibiting muscle‐specific scaling with velocity. This finding aligns with evidence that, once activation exceeds approximately 10% MVC, further increases yield minimal additional stability [41], and that postural tasks often recruit deep and superficial fibres as a single synergy [49,50].

Clinically, the present findings suggest that end‑range trunk‑muscle activation may reach a similar level across 1 s, 3 s, and 5 s lowering velocities; however, mid‑phase control appears to remain velocity‑dependent. Consequently, clinicians might not need to enforce strict stopwatch pacing during routine SCST Level 3 assessment; allowing patients to lower the limb at an approximate 2–3 seconds self‑selected velocity is likely to be adequate in most cases. Slower velocities can still be useful for individuals in early‑phase rehabilitation or those with pain sensitivity, whereas introducing a 1 s lowering phase at a later stage could help train rapid braking capacity without compromising stability. In athletic populations that frequently decelerate the limb quickly, for example during jump landings or sudden directional changes, performing SCST Level 3 at faster velocities may provide a practical drill for rehearsing the front‑loaded trunk‑muscle activation profile observed in this study.

This study has several limitations that may affect the generalizability and interpretation of our findings.

First, our sample comprised only healthy young adult males; therefore, the findings cannot be directly generalized to females, older adults, or clinical populations (e.g., individuals with low back pain). Future research should include these groups to assess whether the present findings generalize and to enhance external validity.

Second, we only analyzed the leg-lowering phase of SCST Level 3. Subsequent studies should incorporate continuous motion analysis across all SCST phases, including higher-intensity levels involving bilateral leg extension or added resistance.

Third, our 250 ms pre-movement analysis window may have lacked sensitivity to capture very brief (tens to hundreds of milliseconds) anticipatory contractions [13–15,17]. Finer time-resolution analysis is needed for more accurate assessment of feedforward activity.

Fourth, while sEMG was used, it is susceptible to crosstalk, especially for deep muscles like IO/TrA [51]. Moreover, excessively synchronized or generalized activation patterns may hinder the nuanced, segment-specific control required in more dynamic tasks [52,53]. Future studies could improve accuracy with needle electrodes or ultrasonography. Additionally, our six-channel EMG montage was not designed for formal muscle-synergy extraction; therefore, future work should expand the EMG montage (e.g., bilateral abdominal wall, erector spinae, multifidus, diaphragm) and apply muscle-synergy analysis using non-negative matrix factorization (NMF).

Fifth, despite using PBUs for lumbopelvic control [35–37], our PBU-based index had limitations. The wide 40 ± 10 mmHg tolerance band may have lacked sensitivity for subtle instabilities, and we did not digitize the PBU output, which prevented pressure–time metrics or variability analyses. Future studies should record the PBU signal continuously and time-synchronize it with EMG and 3-D pelvic/lumbar kinematics to quantify dynamic stability directly.

Sixth, our acceleration sensors alone couldn’t provide detailed 3D measurements of pelvic or thoracic displacement. Integrating motion capture systems with multiple accelerometers would allow for more comprehensive trunk motion analysis.

Seventh, while we controlled electrode placement and instruction consistency, we didn’t formally evaluate intra-rater or inter-trial reliability for EMG outcomes or SCST performance in this sample. This unquantified variability should be considered, despite prior reports of high test-retest reliability for SCST Level 3 [34].

Finally, although velocity conditions were randomized and rest periods provided, we didn’t obtain objective fatigue measures or formally test for order effects. This means subtle cumulative fatigue or order influences, particularly in the fast condition, cannot be ruled out. Future studies should consider longer rest intervals, multiple testing sessions, and explicit fatigue/order-effect analyses.

## Conclusion

This study investigated the effects of different movement velocities (slow, 5 s; medium, 3 s; and fast, 1 s) on trunk muscle activity (IO/TrA, EO, RA) during SCST Level 3 performed in a supine position. The results indicate that movement velocity influences the pattern of muscle activation, particularly during the mid- to late-phase of motion. However, by the end of the task, muscle activity levels converged across all velocity conditions.

Additionally, due to the stable supine posture and the baseline muscle activation required in the initial position, no substantial differences were observed between deep and superficial muscle activity patterns or in left–right asymmetry. Under the fast condition, an early-phase co-contraction strategy (early concentration strategy) was observed, whereas in the slow and medium conditions, muscle activation followed a more gradual increase (late-phase progressive strategy). Despite these differences in activation timing, all conditions ultimately reached comparable levels of trunk muscle activity.

These findings suggest that for routine SCST Level 3 screening, when the main goal is simply to achieve end‑range co‑contraction, a self‑selected lowering velocity of around 2–3 seconds is sufficient and strict stopwatch pacing is not required. For patients in the early phases of rehabilitation or those with pain sensitivity, lowering the limb over 5 seconds provides a gentler stability challenge, whereas higher‑functioning individuals and athletes may benefit from a faster 1 second velocity both to assess rapid trunk‑muscle recruitment and to train dynamic stabilization. Clinicians can therefore adjust SCST Level 3 velocity according to each person’s functional level and the specific objectives of their exercise progression, rehabilitation protocol, or performance screening. Future research should record the PBU pressure signal continuously and time-synchronize it with EMG and 3-D kinematics; expand the multi-muscle sEMG montage to enable muscle-synergy analysis (e.g., NMF), recruit diverse cohorts (females, older adults, and patients, including those with low back pain), and examine functional tasks that tax rapid trunk stabilization (e.g., jump landings and directional changes), alongside 3-D kinematic analysis and externally loaded or higher-level SCST variants.

## Supporting information

S1 Table Trial‑level RMS (µV), MVC (µV), and %MVC values for every participant (ID K10001–K10030) across movement velocities (1 s, 3 s, 5 s), movement phases (pre 500 ms, pre 250 ms, post 1–post 3), muscles (IO/TrA, EO, RA), sides (right, left), and trials (1, 2). (CSV)
